# Second Metatarsal Length and Transfer Ulcers After First Metatarsal Amputation in Diabetic Foot Infections

**DOI:** 10.1177/10711007241232970

**Published:** 2024-03-18

**Authors:** Nicola A. Cavalcanti, Katharina Martini, Tobias Götschi, Nicola Krähenbühl, Madlaina Schöni, Felix W. A. Waibel

**Affiliations:** 1 Department of Orthopaedics, Balgrist University Hospital, Zurich, Switzerland; 2 Department of Radiology, Balgrist University Hospital, Zurich, Switzerland; 3 Institute for Biomechanics, ETH Zurich, Zurich, Switzerland; 4 Department of Orthopaedics, University Hospital Basel, Basel, Switzerland

**Keywords:** metatarsal measurement, transfer ulcers, diabetic foot infections, second metatarsal length

## Abstract

**Background:**

Plantar transfer ulcers (TUs) underneath the second metatarsal head are frequent after first metatarsal ray amputations due to diabetic foot infections. Whether the second metatarsal length (2ML) is associated with TU occurrence in these patients is unclear. This study evaluated whether 2ML is associated with TU occurrence after first-ray amputations and whether ulcer-free survival is shorter in patients with “excess” 2ML.

**Methods:**

Forty-two patients with a mean age of 67 (range 33-93) years, diabetes, and first metatarsal ray amputation (first amputation at the affected foot) were included. Two independent readers measured the 2ML using the Coughlin method. A protrusion of more than 4.0 mm of the second metatarsal was defined as “excess” 2ML. The effect of 2ML on ulcer occurrence was analyzed using a multivariate Cox regression model. A Kaplan-Meier curve for TU-free survival was constructed comparing the 2 groups of “normal” (n = 21) and “excess” 2ML (n = 21).

**Results:**

Interrater reliability was excellent. TUs underneath the second metatarsal occurred in 15 (36%) patients. In agreement with our hypothesis, 2ML was nonsignificantly different in patients with TUs, recording a mean of 5.3 (SD 2.5) mm, compared to patients without 4.0 (SD 2.3) mm (hazard ratio [HR] 1.12, 95% CI 0.89-1.41), whereas insulin dependence was associated with ulcer occurrence (HR 0.33, 95% CI 0.11-0.99).

**Conclusion:**

In our relatively small study population with a cutoff level of 4 mm for excess 2ML, ulcer-free survival was similar in patients with “normal” and “excess” 2ML.

**Level of Evidence:**

Level III, retrospective comparative study.

## Graphical abstract

**Graphical Abstract:**
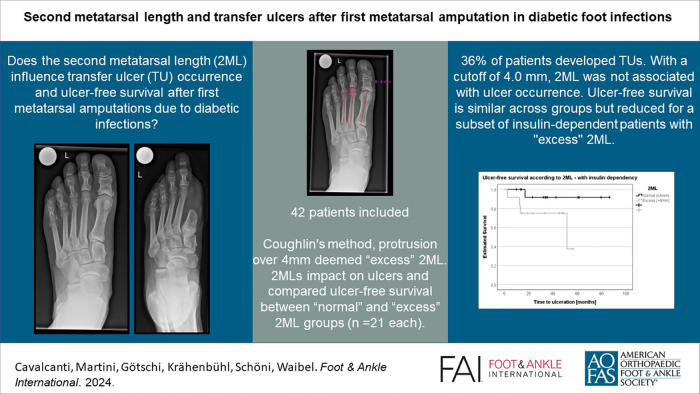
This is a visual representation of the abstract.

## Introduction

Diabetes mellitus (DM) is a multimodal chronic disease that has become one of the most prominent public health issues, affecting 537 million adults worldwide.^28^ Various complications occur in patients suffering from DM.^3,40^ Among these complications, peripheral artery disease, diabetic foot infections, diabetic foot ulcers (DFUs), and amputations are major socioeconomic problems.^2,39^ These issues need improved treatment and, generally, strategies for prevention.^1,2,27,45^ The International Diabetes Federation reported a DFU prevalence of up to 17% in European countries, with recurring DFUs of up to 42%.^10,17,28,42,48^

DFUs often occur underneath the first metatarsal head and require intense treatment.^8,12,44^ In case of infection, treatment frequently leads to partial amputation of the first ray.^5,23,30^ Loss of the first metatarsal head in diabetic foot amputation leads to increased peak plantar pressure beneath the adjacent rays, whereas the second metatarsal head is the only one significantly affected by increased pressure in up to 60% of first-ray amputations.^18^ Transfer ulcers (TUs) can commonly be found in this location and can generally lead to 12% to 60% reamputations.^6,12,21,37,47^ At the same time, relevant prognostic factors have yet to be identified.

Second metatarsal length can be measured by several methods previously assessed by Chauhan et al^9^ for their variability. The three, namely, Coughlin,^11^ Maestro,^34^ and Hardy-Clapham methods,^22^ have been widely described to measure the metatarsal length before surgical interventions, such as osteotomies for the treatment of metatarsalgia.^13,14,24,32,41^ A relative protrusion of the second metatarsal of more than 4 mm compared with the first metatarsal using the Coughlin measurement method has been defined as an excess length in the literature.^9,35^

This study aimed to evaluate whether second metatarsal length (2ML) is associated with TU occurrence after first-ray amputations due to diabetic foot infection overall and whether ulcer-free survival is worse in those patients with “excess” 2ML.

## Material and Methods

Balgrist University Hospital is a tertiary referral hospital for orthopaedic surgery with a specialized unit for the diabetic foot. All patients treated at our hospital are asked to sign a general consent for retrospective data analysis. The study was designed as a retrospective comparative study featuring uni- and multivariate linear analyses and Kaplan-Meier survival curves. This study was approved by the institutional review board and the local ethical committee of Zurich, Switzerland (BASEC-Nr. 2019-01994) prior to the initiation of the study as a retrospective analysis without patient contact.

### Subjects

We searched our retrospective database of diabetic foot amputations (amputations included from January 1, 2000, to March 31, 2020) for patients with amputations of the first metatarsal and identified 79 patients. Inclusion criteria were a minimum clinical follow-up of 12 months, an available weightbearing or nonweightbearing dorsoplantar foot radiograph prior to first-ray amputation, and general consent. Exclusion criteria were missing weightbearing or nonweightbearing dorsoplantar foot radiographs before first-ray amputation, age below 18 years, clinical follow-up of <12 months, and missing general consent. Further, patients with prior amputations to other metatarsals on the same foot were excluded. Prior toe amputation was not considered an exclusion criterion. Only complete data were analyzed. Forty-two patients were eligible for study inclusion. Demographic and clinical data were obtained from the patient records. After first-ray amputation, each patient was provided with custom-made orthopaedic insoles and a stiff rocker sole to prevent the development of further ulcers. Adherence to orthopaedic footwear was not explicitly recorded.

### Measurements

**Figure 1.**
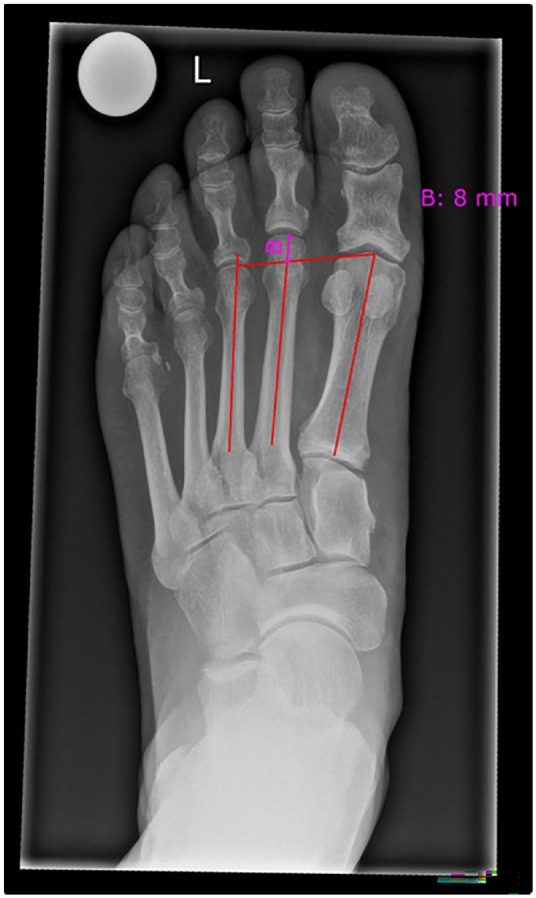
A radiograph of the left foot (dorsoplantar view, weightbearing) of a patient who is to undergo first-ray amputation. Visualization of the Coughlin method marked with red lines and measured in bright pink (“B”). [See online article for color figure.]

Two investigators, an orthopaedic surgery resident (investigator 1, N.A.C.) and a radiology fellow (investigator 2, K.M.), independently performed measurements of the metatarsal length in the MEDICAD planning software (MediCad Multimedia Co, Niedernviehbach, Germany). Investigator 2 was blinded to clinical data and only received the imaging material to avert biased measurements within or between the investigators. Accurate measurements were ensured by scaling the radiographs with a 25-mm reference ball included in the image. If no reference ball was available for scaling, an experienced radiologist defined the accurate scaling by laterally or centrally measuring the distal or medial phalanges of the fourth or fifth toe. The measurement was apprehended in a separately available MRI of the forefoot of the same patient in the institutional PACS program (Phönix PACS GmbH, Freiburg im Breisgau, Germany). After the accurate scaling was ensured, the Coughlin method was applied to measure the relative protrusion or retraction of the second metatarsal, as further described below.^11^ As shown in Figure 1, the Coughlin technique measures the distance from the apex of the articular surface of the second metatarsal to a connecting line between the apex of the articular surface of the first and third metatarsals.^11^

**Figure 2.**
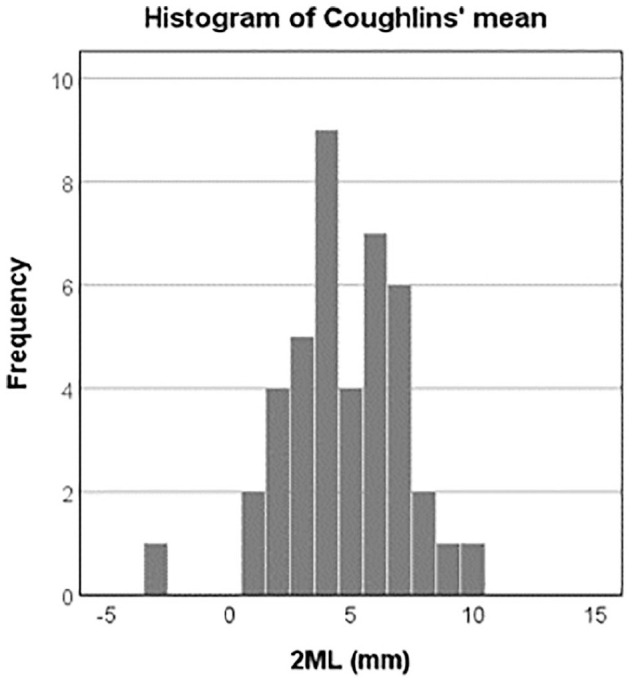
Histogram of the population’s mean second metatarsal length (2ML) according to the Coughlin method. Our cohort reported a mean of 4.5 (SD 2.4) mm for all 42 subjects. The subjects were categorized into a group with a 2ML of ≤4 mm and another group with a 2ML of >4 mm.

As previously suggested by Chauhan et al,^9^ a relative protrusion of the second metatarsal was documented with a positive value, whereas a retraction was documented with a negative value. Our cohort showed a mean of 4.5 (SD 2.4) mm for all 42 subjects. Because the suggested threshold value seemed reasonable according to our available data, we categorized the patients on the basis of the length of the second metatarsal according to the Coughlin method into “normal” or ≤4 mm and relative “excess” length, therefore, >4 mm, as visualized in Figure 2.

**Figure 3.**
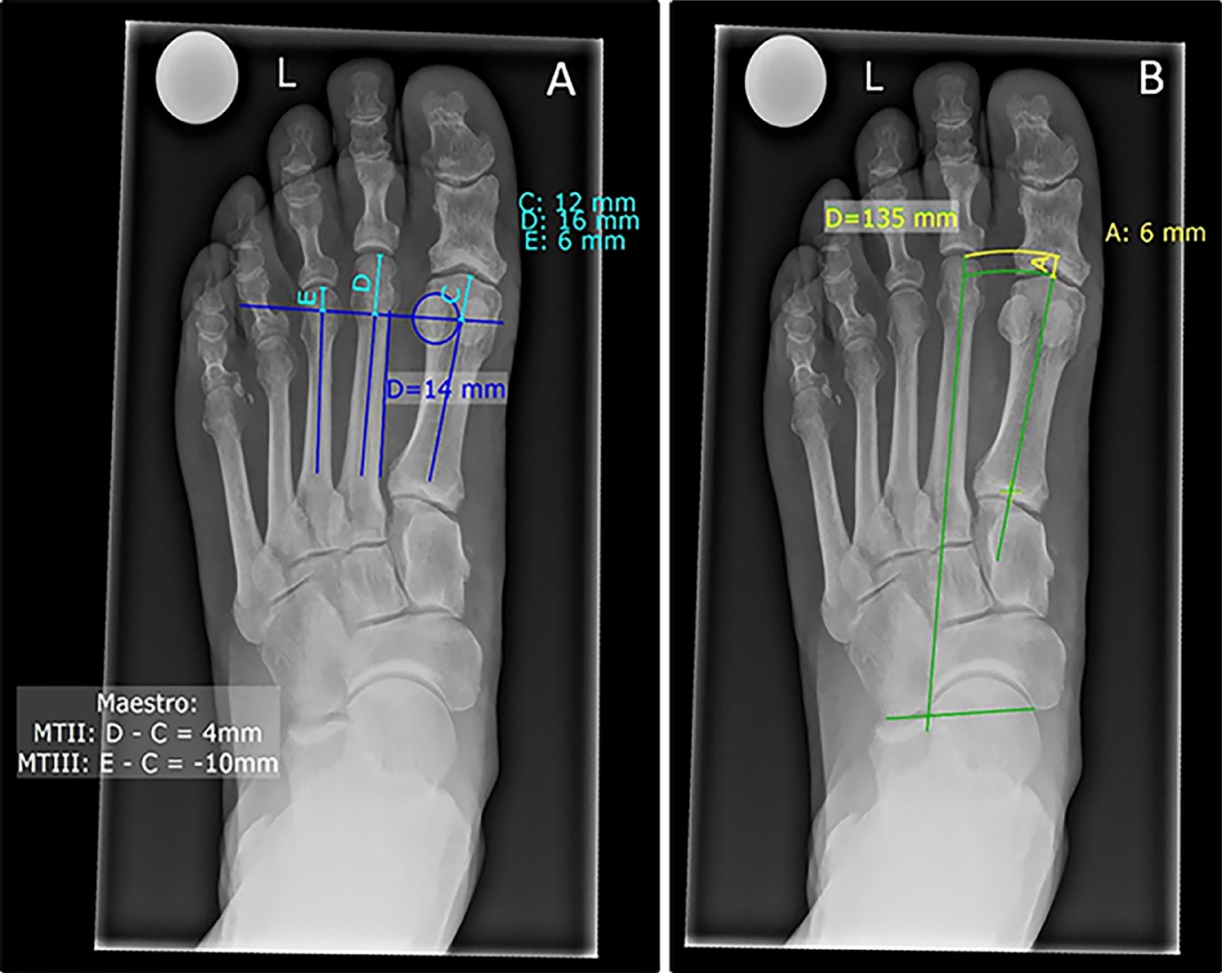
Radiographs of the left foot (dorsoplantar view, weightbearing) of a patient who is to undergo first-ray amputation. (A) Visualization of the Maestro technique marked with blue lines and measured in turquoise (“C, D, E”). (B) Hardy-Clapham technique marked with green lines and measured in yellow (“A”). [See online article for color figure.]

**Figure 4.**
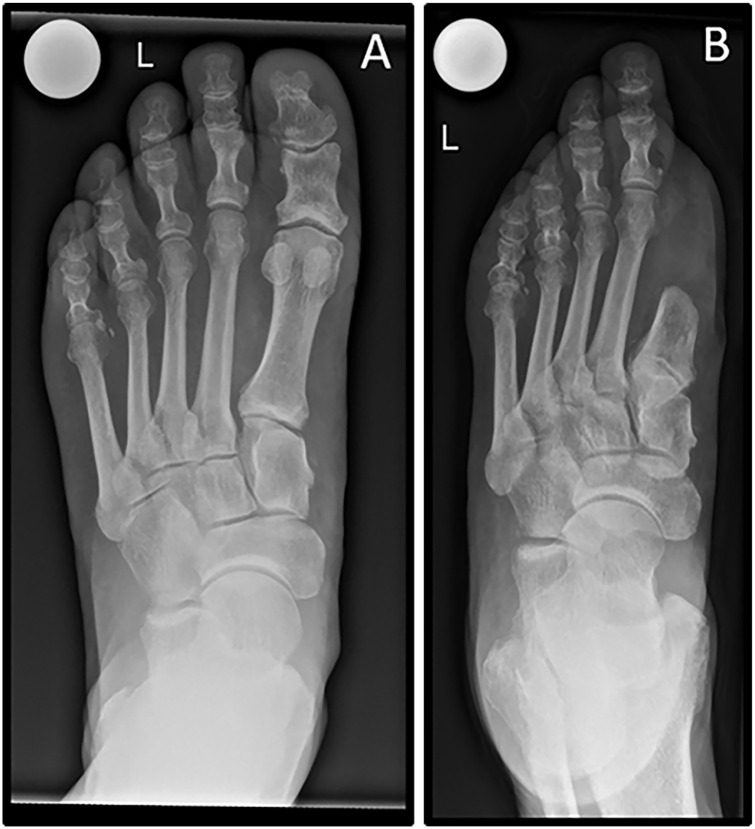
(A) A radiograph of the left foot (dorsoplantar view, weightbearing) of a patient who is to undergo first-ray amputation. (B) Radiograph of the same left foot (dorsoplantar view, nonweightbearing) 2 months after first-ray amputation.

Measurements were then repeated using the methods of Maestro and Hardy-Clapham as there is no agreement in the literature on which 2ML measurement method is preferable.^9,22,34^ The Maestro technique, as seen in Figure 3A, measures the distance from a line perpendicular to the medial border of the second metatarsal, centered in the lateral sesamoid, to each metatarsal tip.^34^ As previously described,^9^ we calculated the relative length of the second metatarsal as a distance to the first (M1-M2) and third (M2-M3) metatarsals. The Hardy-Clapham technique, as seen in Figure 3B, measures the distance of 2 arches at the apex of the first and second metatarsals using a line between the CC joint (calcaneocuboid) and the medial navicular tuberosity.^22^ Figure 4A further illustrates an example of a left foot radiograph (dorsoplantar view, weightbearing) before first-ray amputation and 2 months postoperatively (Figure 4B).

Furthermore, investigator 1 measured the length of the first metatarsal before and after partial amputation to evaluate if the resection length and residual length may have had an influence on the occurrence of TUs in our cohort, as shown by Hong et al.^25^

### Statistical Analysis

Demographical data, Fisher exact tests, and Mann-Whitney *U* tests, as applicable, were used for group comparisons between patients with and without TUs. Interrater reliability for the different measurement methods was quantified by the intraclass correlation coefficient (ICC) based on single measures in a 2-way random effects model. A univariate risk analysis was conducted to identify potential confounders influencing TU occurrence. The effect of the 2ML on time to TU occurrence (months) in the presence of these potential confounders was then assessed using a separate multivariate Cox regression model for all 3 measurement methods and in a similar fashion for the first-ray measurements. Finally, patients were grouped into “normal” 2ML and “excess” 2ML according to the Coughlin method, with a 2ML of more than 4 mm being defined as “excess” 2ML. A Kaplan-Meier survival curve was constructed to compare the 2 groups’ ulcer-free survival. Finally, a demographic analysis and 2 separate Kaplan-Meier survival curves were constructed to compare ulcer-free survival between groups with and without insulin dependence. The statistical analysis was made using SPSS software (version 26; IBM, Armonk, NY) and Stata (version 13.1; StataCorp LP, College Station, TX).

## Results

The mean follow-up was 77 months with a minimum of 12 months. The patients’ mean age at first-ray amputation was 67 (range 33-93) years. After complete data analysis, we included 9 female and 33 male patients and 22 left and 20 right feet. Fifteen patients (36%) developed TUs underneath the second metatarsal head: 5 of 21 (24%) in the “normal” 2ML group and 10 of 21 (48%) in the “excess” 2ML group (*P* = .16; Table 2). Insulin dependence was the only variable significantly associated with ulcer occurrence (*P* ≤ .01; Table 1).

**Table 1.** Patient Demographics.^a^

General Characteristics	Transfer Ulcer		
	No (n =27)	Yes (n =15)	*P* Value
Left side	14 (51.9)	8 (53.3)	>.99
Age, y	69.4 ± 12.0	66.7 ± 9.3	.35 (0.18)
Male sex	20 (74.1)	13 (86.7)	.45
BMI	29.1 ± 5.6	30.3 ± 5.3	.47 (0.22)
Diabetes			
Type 1	2 (7.4)	0 (0.0)	.53
Type 2	25 (92.6)	15 (100.0)	
Oral antidiabetics	13 (48.1)	11 (73.3)	.19
Insulin dependence	22 (81.5)	5 (33.3)	<**.01**
HbA_1c_ at MT1 amputation	8.0 ± 1.6	7.2 ± 1.0	.39 (0.58)
Years of diabetes until MTI amputation	17 ± 10	14 ± 7	.42 (0.34)
Polyneuropathy	24 (88.9)	14 (93.3)	>.99
CKD	15 (55.6)	5 (33.3)	.21
CKD stages			
1-4	13 (48.1)	4 (26.7)	.32
5	2 (7.4)	1 (6.7)	
CAD	9 (33.3)	5 (33.3)	>.99
PAD	21 (77.8)	10 (66.7)	.48
PAD stages (Fontaine classification)			
1	10 (37.0)	3 (20.0)	.72
2	4 (14.8)	4 (26.7)	
3	5 (18.5)	2 (13.3)	
4	2 (7.4)	1 (6.7)	
Angioplasty	12 (44.4)	9 (60.0)	.52
Immunosuppressants	0 (0.0)	1 (6.7)	.36
Active nicotine dependence	4 (14.8)	5 (33.3)	.24
Alcohol dependence	4 (14.8)	5 (33.3)	.24
Follow-up duration, mo	45.3 ± 25.8	82.9 ± 67.7	.07 (0.92)

Abbreviations: BMI, body mass index; CAD, coronary artery disease; CKD, chronic kidney disease; HbA_1c_, hemoglobin A_1c_; MT1, metatarsal 1; PAD, peripheral artery disease.

a Data are shown as n (%) or mean ± SD. Fisher exact test identifies insulin dependence as the cohort’s only significant factor for transfer ulcer development. *P* values are shown with the Cohen effect size (*d*) when applicable. Boldface indicates significance (*P* < .05).

### Measurements and Reliability

Measurement results for each method are demonstrated in Table 2. All measurement methods showed excellent interrater reliability. The ICC was 0.93 (95% CI 0.88-0.96) for the Coughlin method, 0.98 (95% CI 0.95-0.99) for the Hardy-Clapham method, and 0.96 (95% CI 0.94-0.98) for the Maestro method. Regarding the risk of TU development, the Coughlin method showed a mean 2ML of 5.3 (SD 2.5) mm with and 4.0 (SD 2.3) mm without ulcer occurrence (*P* = .16). Hardy-Clapham showed a mean 2ML of 4.3 (SD 6.8) mm and 2.7 (SD 4.1) mm (*P* = .33), respectively. The Maestro mean 2ML recorded 2.4 (SD 7.1) mm with and 0.8 (SD 4.0) mm without TU development (*P* = .16). None of the 2ML measurement methods showed significance. After partial first-ray amputation, our cohort showed a mean residual length of 29.1 (SD 15.5) mm with and 32.9 (SD 9.4) mm without later TU occurrence. However, there was no significant difference between the residual first metatarsal with and without TU development.

**Table 2.** Metatarsal Length and Transfer Ulcer Development.^a^

Measurement Methods	Transfer Ulcer		
	No (n =27)	Yes (n =15)	*P* Value
Coughlin mean, mm	4.0 ± 2.3	5.3 ± 2.5	.16 (0.55)
Hardy-Clapham mean, mm	2.7 ± 4.1	4.3 ± 6.8	.33 (0.32)
Maestro mean, mm	0.8 ± 4.0	2.4 ± 7.1	.16 (0.31)
MT1 residual length, mm	32.9 ± 9.4	29.1 ± 15.5	.46 (0.33)
MT1 resection length, mm	31.6 ± 10.0	38.2 ± 17.1	.5 (0.53)
Preservation of <1/3 MT1 residual length	1 (5.0)	3 (23.1)	.29

Abbreviation: MT1, metatarsal 1.

a The association of the first and second metatarsal measurement with the effect of the risk of transfer ulcer development. Data are shown as number (%) or mean ± SD. *P* values are shown with Cohen effect size (*d*) when applicable.

### Multivariate Analysis

Each measurement method had no significant association between 2ML and TU occurrence in our cohort. Likewise, insulin dependence was a risk factor for ulcer occurrence in each measurement method. The results are summarized in Table 3.

**Table 3.** Multivariate Cox Regression Analyses.^a^

	HR	95% CI
Coughlin		
Second metatarsal length	1.12	0.89-1.41
Insulin dependence	0.33	0.11-0.99
Hardy-Clapham		
Second metatarsal length	1.07	0.97-1.18
Insulin dependence	0.29	0.09-0.88
Maestro		
Second metatarsal length	1.07	0.97-1.18
Insulin dependence	0.29	0.10-0.89
MT1 resection	1.01	0.98-1.04
Insulin dependence	0.23	0.06-0.87
Preservation of <1/3 MT1 residual length	0.58	0.12-2.77
Insulin dependence	2.98	0.65-13.55

Abbreviations: HR, hazard ratio; MT1, metatarsal 1; TU, transfer ulcers.

a Multivariate Cox regression analyses test the association of time to transfer ulcer occurrence (months), second metatarsal length, and insulin dependence, which was identified as the only significant potential confounder of TU occurrence in the previously conducted univariate risk analysis.

### Ulcer-Free Survival and Insulin Dependence

**Figure 5.**
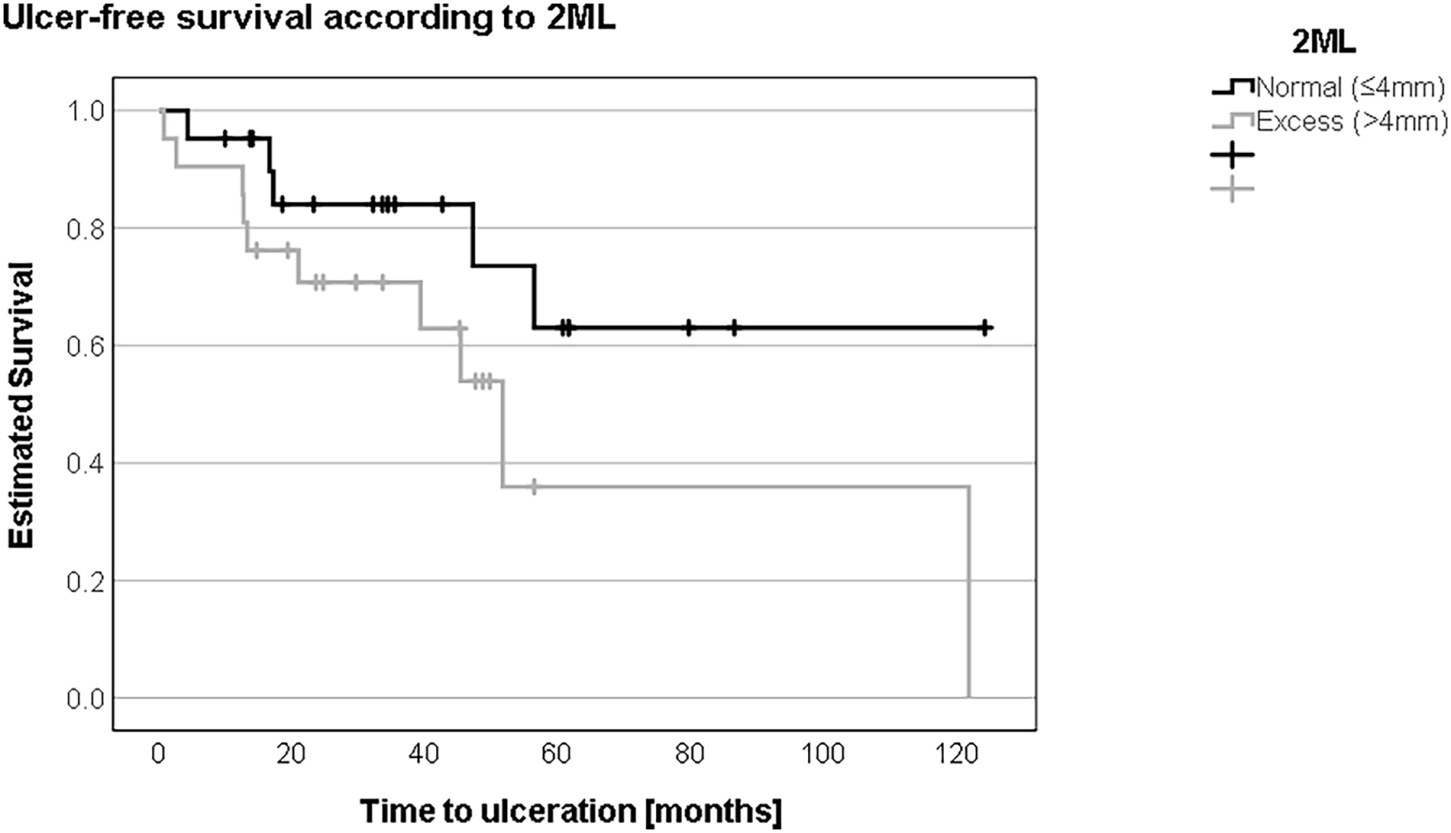
Grouping patients according to the second metatarsal length (2ML) using the Coughlin method into “normal” or ≤4 mm and “excess” length or >4 mm, ulcer-free survival was shorter in patients with excess length (63 vs 91 months).

Regarding demographic data (Table 4), no significant differences existed in the insulin-dependent and independent cohorts with or without TU occurrence. According to the Coughlin method, the Kaplan-Meier survival curve (Figure 5) showed no statistically significant difference in ulcer-free survival between patients with “normal” 2ML and “excess” 2ML (63.4 months [SD 14.8, 95% CI 34.4-92.3] vs 91.3 months [SD 12.2, 95% CI 67.3-115.2]; log-rank test: *P* = .09).

**Table 4.** Transfer Ulcer Development With and Without Insulin Dependence.^a^

	Transfer Ulcer					
	No			Yes		
	Insulin Dependence			Insulin Dependence		
General Characteristics	No (n =5)	Yes (n =22)	*P* Value	No (n =10)	Yes (n =5)	*P* Value
Side (left)	2 (40.0)	12 (54.5)	.65	6 (60.0)	2 (40.0)	.61
Age, y	73 ± 14	68 ± 12	.7 (0.40)	64 ± 8	73 ± 8	.07 (1.13)
Male sex	3 (60.0)	17 (77.3)	.58	9 (90.0)	4 (80.0)	1
Diabetes						
Type 1	0 (0.0)	2 (9.1)	1	0 (0.0)	0 (0.0)	1
Type 2	5 (100.0)	20 (90.9)		10 (100.0)	5 (100.0)	
Oral antidiabetics	4 (80.0)	9 (40.9)	.17	7 (70.0)	4 (80.0)	1
HbA_1c_ at MT1 amputation	6.4 ± 0.2	8.2 ± 1.6	**.04** (1.34)	7.3 ± 1.3	7.2 ± 0.5	.93 (0.10)
Years of diabetes until MT1 amputation	7 ± 3	19 ± 9	**.04** (1.52)	12 ± 7	16 ± 5	.44 (0.63)
Follow-up duration, mo	59.6 ± 40.3	42.1 ± 21.4	.41 (0.70)	100.9 ± 75.5	47.1 ± 28.2	.21 (0.90)

Abbreviations: HbA_1c_, hemoglobin A_1c_; MT1, metatarsal 1.

a Analysis of the transfer ulcer development regarding patients dependent on insulin. Data are shown as n (%) or mean ± SD. *P* values are shown with Cohen effect size (*d*) when applicable. Boldface indicates significance (*P* < .05).

**Figure 6.**
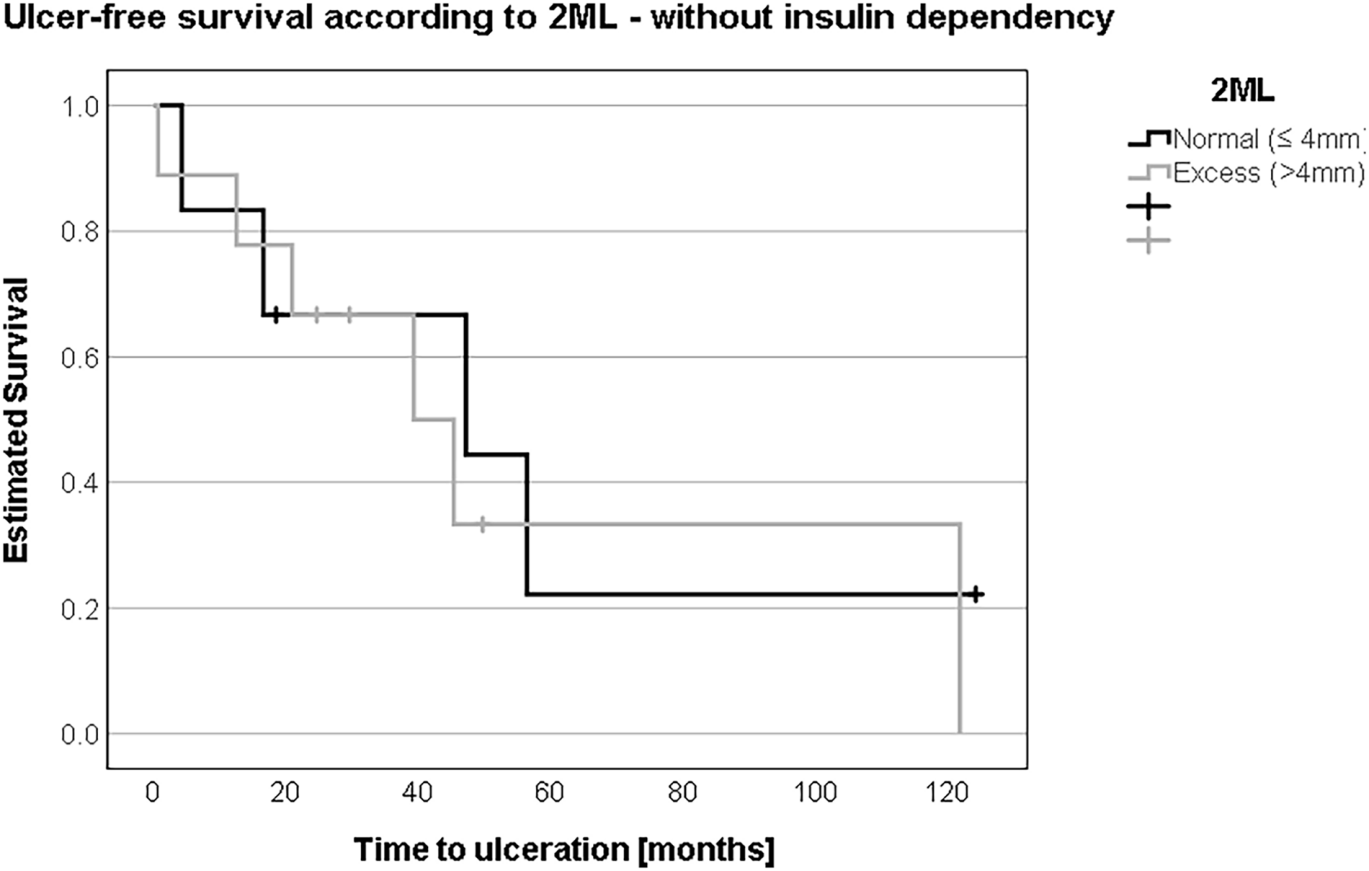
Without insulin dependence, there was no relevant difference in the development of transfer ulcers between the “normal” or less than/equal to 4 mm and “excess” or more than 4 mm groups in second metatarsal length (2ML), notably 54 vs 58 months.

**Figure 7.**
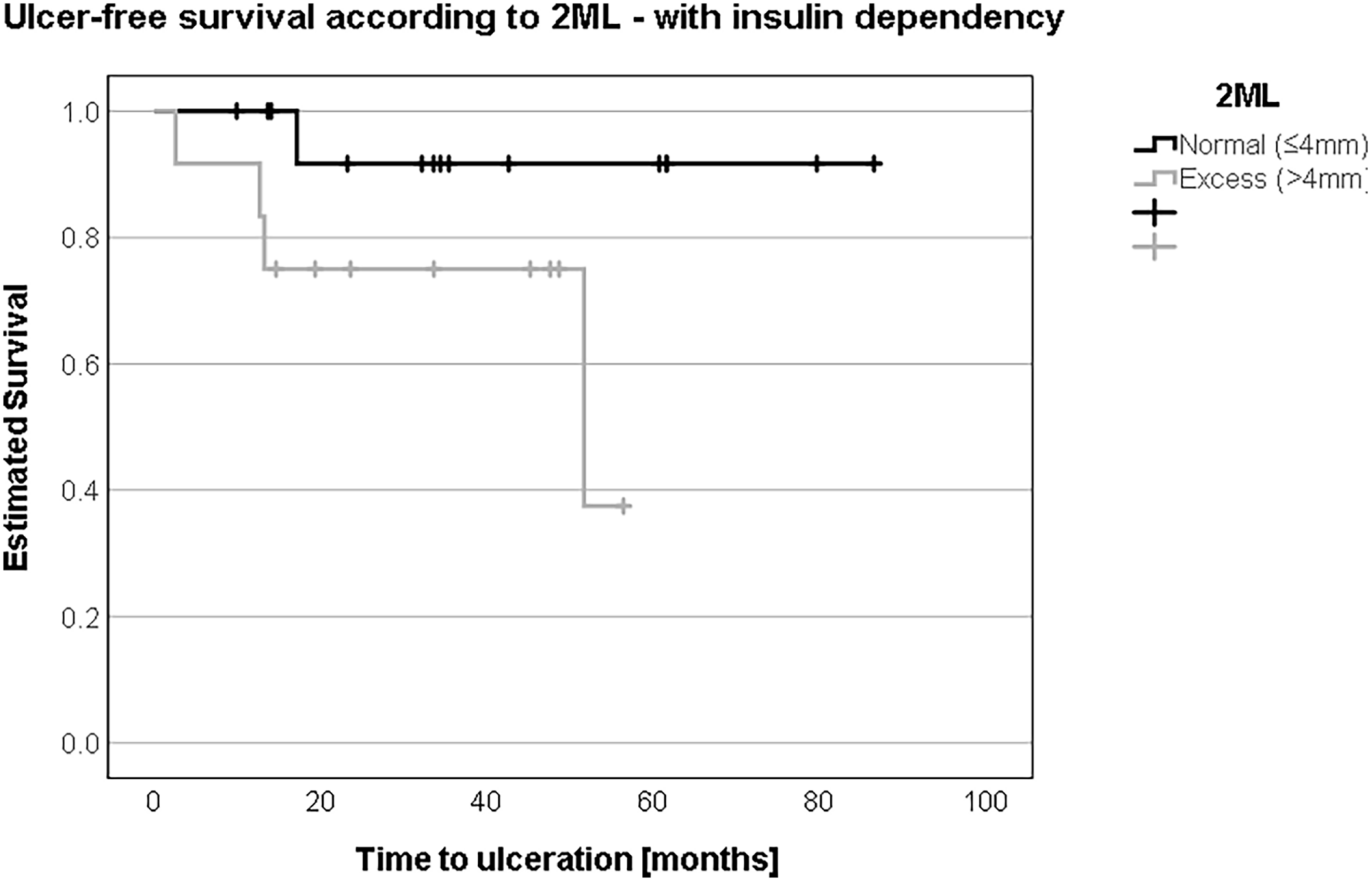
In the presence of insulin dependence, an “excess” or more than 4 mm in second metatarsal length (2ML) represented a substantially shorter ulcer-free survival at 42 vs 80 months.

In patients without insulin dependence, ulcer-free survival was not different comparing patients with “normal” 2ML to patients with “excess” 2ML according to the Coughlin method (54.1 months (SD 18.3, 95% CI 18.2-90.0) vs. 58.5 months (SD 19.0, 95% CI 21.2-95.8); log-rank test: *P* = .69) (Figure 6). In insulin-dependent patients, ulcer-free survival tended to be shorter in patients with “excess” 2ML compared to patients with “normal” 2ML (42.9 months (SD 5.8, 95% CI 31.6-54.2) vs. 80.8 months (SD 5.5, 95% CI 69.9-91.6); log-rank test: *P* = .06) (Figure 7).

## Discussion

With the numbers available we found no association of 2ML with TU development underneath the second metatarsal head in patients with prior first metatarsal amputation due to diabetic foot infections. Insulin dependence was associated with a lower risk of TU occurrence in our cohort, although the 95% confidence interval (0.11-0.99) and the small sample size suggests this finding should be explored further in a larger population. In contrast, patients had considerably shorter ulcer-free survival when insulin dependence was stratified into “normal” and “excess” 2ML.

The literature reports that the relative length of the second metatarsal (compared to the third metatarsal) is a key predictor of the ratio of peak pressure, pressure-time integral, and force-time integral for the second vs third metatarsal.^16^ It is also reported that the second metatarsal with an excess of 2ML will likely experience higher loads, which can lead to injury.^16^ Overall, our series could positively associate a tendency for “excess” 2ML with TUs. However, “excess” 2ML, measured by the Coughlin method, was not statistically significantly associated with TUs. As the literature suggests a threshold value of more than 4.0 mm as the excess length of the second metatarsal if measured with the Coughlin method,^9,35^ we constructed a Kaplan-Meier survival estimate for patients with and without excess 2ML. We failed to demonstrate any significant difference in TU-free survival between the 2 groups. Multivariate analysis showed a positive association between TU occurrence and 2ML for the Maestro and Hardy-Clapham methods. Nonetheless, statistical significance could not be confirmed for either method.

Possible anatomical reasons for TUs and reulcerations after first-ray amputations have been investigated before. One group described that the shorter the protrusion of the first metatarsal with respect to the longest of metatarsals second to fifth, the higher the probability for reulceration in case of prior metatarsal head resection.^36^ Although closest to our study question, it was not limited to first metatarsal amputations or second metatarsal measurements, making a direct comparison with our study impossible. Another study analyzing reulcerations after metatarsal head resections in diabetic foot osteomyelitis found that a calculated metatarsal resection rate of less than 25% was associated with the development of a recurrence after surgery in the midterm follow-up.^38^ This study was not limited to first metatarsal amputations, making comparison unfeasible.

Other research has focused on the residual length of the first metatarsal after amputation. One study measured the remaining length of the first metatarsal after the first metatarsal amputation and found that a loss of 37 mm of first metatarsal length was associated with a 9 times greater likelihood of ulceration. Measurement of 2ML was not part of their study design.^23^ Further, preservation of more than one-third of the first metatarsal length compared with less than one-third after first ray amputation was associated with a lower likelihood of transfer amputation of the lesser toes.^25^ Our cohort did not show significance between the occurrence of TU and increased residual first metatarsal length.

We report reduced occurrence of transfer ulcerations in insulin-dependent patients, provided that patients were not stratified for “normal” and “excess” 2ML. This finding may be due to the potential healing benefits of insulin on DFUs, as previously reported in the literature.^15,19,20,26,29,43^ Possible explanations are the capability of insulin to stimulate cell migration and to accelerate epithelialization.^26,33^ Additionally, literature shows that glycemic control can decrease the likelihood of lower extremity amputations and the long-term DFU risk.^7,31^ With the numbers available to us, age, sex, diabetes type, oral diabetics, HbA_1c_, and the time from first diagnosis of diabetes to partial first-ray amputation could not be identified as significant risk factors of TU occurrence, with the *P* values of HbA_1c_ and years of diabetes being considered with caution because of the limited sample size. However, the effect of HbA_1c_, glucose levels, and other factors on wound healing should be investigated further in larger patient populations.^31,46^

It is essential to acknowledge the limitations of our research, such as the retrospective study design with its concomitant confounding and selection bias possibilities. Further, a post hoc calculation proposed a minimal sample size of 72 patients; our study is underpowered to claim no significant difference between groups. For this reason, our results must be interpreted with care, so we recommend testing our study’s results on a larger patient population. There could also have been bias with the choice of our measurement methods or cutoff level. In our study, the relative excess length of the second metatarsal was analyzed with the methods described, whereas Bhutta et al^4^ showed that additionally, an increasing hallux valgus angle could decrease the functional 2ML when using the Maestro and Hardy-Clapham methods.

## Conclusions

In this study, “excess” second metatarsal length as we defined it was not associated with TU occurrence after first-ray amputation in diabetic foot infections. Ulcer-free survival was reduced in the small subset of patients with insulin-dependent patients with “excess” 2ML; however, the findings are preliminary and clearly further study in larger cohorts is needed.

## Supplemental Material

sj-pdf-1-fai-10.1177_10711007241232970 – Supplemental material for Second Metatarsal Length and Transfer Ulcers After First Metatarsal Amputation in Diabetic Foot Infections Supplemental material, sj-pdf-1-fai-10.1177_10711007241232970 for Second Metatarsal Length and Transfer Ulcers After First Metatarsal Amputation in Diabetic Foot Infections by Nicola A. Cavalcanti, Katharina Martini, Tobias Götschi, Nicola Krähenbühl, Madlaina Schöni and Felix W. A. Waibel in Foot & Ankle International
